# Imaging features and clinical value of ^18^F-FDG PET/CT for predicting airway involvement in patients with relapsing polychondritis

**DOI:** 10.1186/s13075-023-03156-x

**Published:** 2023-10-14

**Authors:** Jing-Wei Yi, Jun-Feng Huang, Peng Hou, Zi-Kai Lin, Jin-Sheng Lin, Si-Yan Lin, Min Wang, Shi-Yue Li, Xin-Lu Wang

**Affiliations:** 1 Department of Nuclear Medicine, the First Affiliated Hospital of Guangzhou Medical University, Guangzhou, Guangdong China; 2 Guangzhou Institute of Respiratory Health, State Key Laboratory of Respiratory Disease, National Clinical Research Center for Respiratory Disease, National Center for Respiratory Medicine, the First Affiliated Hospital of Guangzhou Medical University, Guangzhou, Guangdong China; 3 Guangzhou Medical University, Guangzhou, Guangdong China; 4 Department of Radiology, Yangchun People’s Hospital, Guangdong, China; 5 The Hospital of Integrated Chinese and Western Medicine of Hunan Province, Changsha, Hunan China

**Keywords:** Relapsing polychondritis, ^18^F-FDG PET/CT, Airway involvement, Imaging feature, Prediction

## Abstract

**Background:**

The clinical value of ^18^F-fluorodeoxyglucose (FDG) positron emission tomography/computed tomography (PET/CT) in assessing relapsing polychondritis (RP) with airway involvement remains controversial. This study aimed to investigate PET/CT features of RP with airway involvement and explore its clinical value in predicting disease pattern, severity and prognosis.

**Methods:**

RP patients with airway involvement who underwent PET/CT from January 2010 to July 2022 were retrospectively reviewed. PET/CT features were analyzed both visually and semiquantitatively with the maximum standardized uptake value (SUVmax) and total lesion glycolysis (TLG). Patterns of airway involvement on PET were summarized. Correlations of SUVmax and TLG of the airway were made with spirometric indicators and serological inflammatory markers (CRP and ESR). In addition, long-term follow-up was conducted through questionnaires in regard to symptom control, subjective feeling, pulmonary function, and quality of life.

**Results:**

Fifty-two cases were finally included. ^18^F-FDG PET showed FDG-avid lesions with increased FDG uptake in the airway among 94.2% of the patients. Three patterns (focal, multifocal and diffuse patterns) were identified. TLG of the whole airway was lower in patients with previous therapy (*p* = 0.046). Bronchoscopy was more sensitive in detecting tracheal abnormalities (90.7% vs.53.5%, *p* = 0.039) but less sensitive for peripheral airway lesions (65.1% vs. 79.1%, *p* = 0.046) compared with PET. SUVmax and TLG of the airway positively correlated with spirometry indicators (FEV1%pred, FEV1/FVC, MEF 50%pred, etc.) and serological inflammatory markers. Five patients died during the follow-up, with two deaths related to airway problems. Higher FDG uptake predicted worse subjective feeling, but not with symptom control or pulmonary function.

**Conclusion:**

PET/CT is a valuable tool for RP with airway involvement, particularly in assessing peripheral airway lesions, and PET/CT related parameters are significantly associated with disease patterns, severity, and long-term outcomes.

**Supplementary Information:**

The online version contains supplementary material available at 10.1186/s13075-023-03156-x.

## Background

Relapsing polychondritis (RP) is a rare, multisystem autoimmune disease of unknown etiology. It is characterized by recurrent inflammation of cartilaginous tissue and proteoglycan-rich structures throughout the body including the ear, nose and airway, etc. [1, 2]. Up to 20-50% of patients will develop respiratory tract involvement, which is considered a poor prognostic factor [3–5]. However, the nonspecific respiratory symptoms of airway involvement are commonly misdiagnosed with asthma, amyloidosis or chronic bronchitis at the initial evaluation [6, 7]. This may lead to delays in diagnosis and treatment, as well as potentially life-threatening complications [5, 8]. Generally, chest CT and pulmonary function tests (PFTs) are the primary modalities to detect airway involvement in patients with RP, but their sensitivity in diagnosing and monitoring the disease appears to be limited [9]. Hence, early and accurate identification of airway involvement is crucial for the management and improved prognosis of patients with RP, and more effective approaches for evaluation and prediction need to be developed.

Fluorine-18 fludeoxyglucose (^18^F-FDG) positron emission tomography/computed tomography (PET/CT) has been widely used in assessing various systemic rheumatic diseases. Previous case reports or series have shown a promising value of ^18^F-FDG PET/CT in the management of RP, including diagnosis, evaluating disease activity, and predicting therapeutic response, especially for patients with airway involvement [10–16]. The typical PET/CT performance of involved airways may present as symmetrically increased FDG uptake [13, 15, 17], thickening of the wall in the laryngo-tracheo-bronchial tree, and the radioactivity might decrease after effective glucocorticoids treatment [11, 15, 16]. Despite its potential advantages, the performance and application of PET/CT for RP with airway involvement remain controversial. For instance, a review of 26 published cases indicated a considerably wide range of SUVmax in the airway, even in patients without treatment, which may increase diagnostic uncertainty [15]. In addition, another study also showed no correlation between SUVmax and the serological inflammatory markers erythrocyte sedimentation rate (ESR) or C-reactive protein (CRP) [14]. Overall, the current evidence with limited sample sizes does not allow for a thorough understanding of the role of PET/CT in RP.

Therefore, this study aimed to comprehensively investigate the clinical and imaging features (especially in PET/CT) of patients with airway involvement of RP based on a retrospective cohort, and further explore the actual value of PET/CT in predicting disease pattern, severity/activity and prognosis.

## Methods

### Study design and subject selection

We retrospectively evaluated patients diagnosed with RP with airway involvement who underwent PET/CT (*n* = 54) from January 2010 to July 2022 at the First Affiliated Hospital of Guangzhou Medical University, Guangdong, China. According to the McAdam’s, Damiani and Levine’s or Michet’s criteria [3, 8, 18, 19], the diagnosis of RP was made based on the combination of clinical features (i.e. auricular, nasal, or respiratory tract chondritis, etc.), histological confirmation and response to corticosteroids or dapsone Airway involvement was evaluated by pulmonologists through carefully reviewed medical records including respiratory symptoms, laryngo- or bronchoscopic results, PFTs, and imaging examinations including CT, MR and PET/CT. Patients without intact Digital Imaging and Communications in Medicine (DICOM) imaging were excluded (*n* = 2). The study gained the approval of our institutional review board, and informed consent was waived for this retrospective study analysis.

### Clinical evaluation and follow-up

The extraction of clinical data includes main symptoms, previous treatment, laboratory tests, pulmonary function tests and laryngo- or bronchoscopic results. The data collected were all obtained within 14 days of the PET/CT scan. Laboratory tests mainly included the complete blood count and inflammatory markers such as ESR and CRP. In addition, other rheumatic-related items, including rheumatoid factors, myeloperoxidase, proteinase 3, and anti-nuclear antibody, were also collected. Indicators of pulmonary function tests were reviewed with five grades (mild, moderate, moderately severe, severe and very severe) as proposed by ATS/ESR [20] as well as four grades (mild, moderate, severe and very severe) according to the GOLD grade system [21]. All laboratory tests and pulmonary function tests were taken after admission and before specific treatment of RP.

Laryngo- or bronchoscopy was considered positive when any of the following signs were present: the disappearance of cartilages, cartilage collapse, airway stenosis, and other abnormal mucosa. Representative images were shown in Fig. S1 (Additional file 1). The results were recorded for different areas, including the larynx, trachea, left/right main bronchi with bronchus intermediate and left/right lobar-segmental bronchi. In this study, the whole airway refers to the airways from the larynx to segmental bronchi, while the central airways refer to the trachea, the main bronchi/bronchus intermediate, and the peripheral airways indicate the lobar-segmental bronchi.

A follow-up questionnaire was designed to evaluate the outcomes of the patients. The questionnaire comprised four parts with a total of 12 questions to evaluate patients’ respiratory symptoms, pulmonary function and quality of life, using St George’s Respiratory Questionnaire (SGRQ), modified Medical Research Council (mMRC) dyspnea scale, Visual Analogue Scale (VAS) and WHO Quality of Life (QoL) scale as references. Respiratory symptoms were assessed based on neck pain, hoarseness, cough and dyspnea. The mMRC scale and VAS were used to evaluate dyspnea and airway symptoms, respectively. QoL was evaluated based on the mental health, appetite and sleep conditions of the patients, as well as their confidence about the disease in the future. The total score was calculated by summing the scores of each part. Additional file 2 shows the specific questions included in this questionnaire. Patients who scored less than 8 in the respiratory symptoms section were considered to have well controlled the symptoms, while scores less than 3 for mMRC represented relatively good pulmonary function. A subjective feeling score of 5 or less was considered indicative of good overall conditions.

### Protocol of PET/CT scans

Patients need to fast for at least 6 h and blood glucose levels should be controlled to less than 11.1 mmol/L. A dose of 3.70-5.55 MBq/kg ^18^F-FDG was administered intravenously. Approximately 60 min after ^18^F-FDG injection, a whole-body CT scan from the base of the skull to mid-thigh was performed, followed by a whole-body PET with the same range. Two PET/CT scanners—Discovery ST 8, GE Healthcare, WI, USA (*n* = 46) and Discovery MI, GE Healthcare, WI, USA (*n* = 6)—were used to perform all the acquisitions with the same procedures. The acquisition parameters were as follows: 140 kV, 150 mAs, pitch 1.675, 512 × 512 image matrix, slice thickness of 3.75 mm, and a total of six or seven cradle positions with 3.5 min/ cradle position for whole-body acquisition and 120 kV, 150 mA; slice thickness, 2.5 mm/ 1.25 mm for Discovery ST 8 and Discovery MI.

### Image analysis

Two board-certified nuclear medicine physicians independently interpreted the initial PET/CT results visually. All disagreements were resolved by consensus with a third board-certified researcher. Details about imaging analysis were summarized in Table S1 (Additional file 3). Briefly, the laryngo-tracheo-bronchial tree was divided into four segments as mentioned above and was classified into 3 patterns according to the number of positive segments on PET: focal (1 positive segment), multifocal (2-3 positive segments), and diffuse (4 positive segments) pattern. The 3D slicer (Version 4.0.1) software was used to measure the standardized uptake value (SUVmax) and total lesion glycolysis (TLG) (Fig. S2, Additional file 4). CT components including destruction of laryngeal cartilages, soft-tissue swelling, tracheo-bronchial stenosis, airway wall thickening and calcification were also analyzed.

### Statistical analysis

Descriptive statistics are presented with the mean ± standard deviation or the median (interquartile range [IQR]). Categorical variables are shown as absolute and relative frequencies. The chi-square test was applied to compare the frequency in different groups. Nonparametric Mann–Whitney U tests were used to compare quantitative continuous data. Spearman’s rank correlation coefficient (*rs*) was calculated to examine the correlations between PET/CT parameters and other clinical data. All tests were two-sided, and *p* values of 0.05 or less denoted statistical significance. All analysis was performed using R Studio (version 4.2.0) and SPSS 20.0 (SPSS Inc., Chicago, IL, USA).

## Results

### Clinical characteristics of patients with airway involvement of RP

Fifty-two RP patients with airway involvement (33 males and 19 females, mean age 46 ± 11.3 years) were finally enrolled in the study. All patients complained of respiratory symptoms, including cough (47, 90.4%), shortness of breath (40, 76.9%), excessive sputum (31, 59.6%), hoarseness (11, 21.2%), chest pain (7, 13.5%) and sore throat (5, 9.6%). Thirteen (25.0%) patients had fever. Twenty-six patients (50.0%) had only respiratory symptoms with/without fever. The median disease duration was 8.5 (IQR: 3.25-12) months. Thirteen patients underwent corticosteroid treatment before PET/CT. The median highest dose of corticosteroids was 40 mg/d and the median duration was 158 days. Immunosuppressive drugs including methotrexate (MTX) and cyclophosphamide (CTX) were taken by 3 and 1 patient, respectively. Details were shown in Table S2 (Additional file 5). ESR and CRP were increased in 76.7% and 85.7% of patients, respectively. Airflow obstruction was observed in 91.7% of patients (33/36). 63.5% (33/52) of patients were diagnosed through biopsy, including auricles (28/31, 75.7%), nasal septum(1/2, 50.0%) and tracheobronchial tree (4/36, 11.1%), the others were clinically diagnosed. Detailed clinical characteristics of patients were listed in Table 1.

**Table 1:** The clinical characteristics of patients

**Characteristics**	**RP patients with airway involvement**
**Number of patients**	52
Treated	13 (25.0%)
Untreated	39 (75.0%)
**Age (year)**^**a**^	48 [37.75-54.25]
**Course of disease (month)**^**a,b**^	8.5 [3.75-12]
**Sex**	
Male	33 (63.5%)
Female	19 (36.5%)
**Main symptoms**	
Cough	47 (90.4%)
Excessive sputum	31 (59.6%)
Shortness of breath	40 (76.9%)
Sore throat	5 (9.6%)
Hoarseness	11 (21.2%)
Chest pain	7 (13.5%)
Fever	13 (25.0%)
**Commorbidities**	9 (17.3%)
Malignancy	2 (22.2%)
Prostate cancer	1 (11.1%)
Non-Hodgkin lymphoma	1 (11.1%)
Other autoimmune diseases	3 (33.3%)
EGPA	2 (22.2%)
Hashimoto’s throiditis	1 (11.1%)
Nodular goiter	2 (22.2%)
Metabolic disease	2 (22.2%)
**Serological index**	
**Inflammatory markers**^**a**^	
ESR (mm/h)	39.5 [20-59] (*n* = 44)
CRP (mg/L)	1.24 [0.29-7.75] (*n* = 40)
**Complete blood count**^**a**^	
WBC (10^9/L)	9.54 [7.69-12.33] (*n* = 48)
Neutrophil (%)	71.75 [63.3-78.8] (*n* = 48)
Platelet (10^9/L)	254 [134-342.25] (*n* = 46)
Hemoglobin (g/L)	113 [105-120.5] (*n* = 47)
**Autoimmune markers**	
RF ( +) (*n* = 41)	0 (0.0%)
MPO ( +) (*n* = 37)	1 (2.7%)
PRO-3 ( +) (*n* = 37)	1 (2.7%)
ANA ( ±) (*n* = 39)	5 (12.8%)
**Lung function tests**	36 (69.2%)
**Five grades to evaluate airway obstruction**	36 (69.2%)
Normal	1 ( 2.8%)
Mild	4 (11.1%)
Moderate	5 (13.9%)
Severe	13 (36.1%)
Very severe	13 (36.1%)
**GOLD system**	21 (58.3%)
Stage I	0 (0.0%)
Stage II	10 (47.6%)
Stage III	7 (33.3%)
Stage IV	4 (19.0%)
**Bronchoscopy**	43 (82.7%)
Positive	43 (100%)
**Methods of diagnosis**	
Auricular biopsy (*n* = 37)	28 (75.7%)
Bronchoscopic biopsy (*n* = 36)	4 (11.1%)
Biopsy of nasal septum (*n* = 2)	1 (50.0%)
Clinically	19 (36.5%)

*EGPA* Eosinophilic granulomatosis with polyangiitis, *ESR* Erythrocyte sedimentation rate, *CRP* C-reactive protein, *WBC* White blood cell, *RF* Rheumatoid factor, *MPO* Myeloperoxidase, *PRO-3* proteinase 3, *ANA* Anti-nuclear antibody

^a^All continuous variables are described with median and interquartile range (IQR)

^b^from the beginning of the symptoms to the day of diagnosis of RP

### Imaging characteristics of airway involvement on PET/CT

All patients presented with airway symptoms, and among the 26 patients who only reported respiratory symptoms, PET/CT revealed extrathoracic abnormalities besides the laryngo-tracheo-tree in 16/26 (61.5%) of patients, including the auricular (7/26, 26.9%), nasal cartilages (6/26, 23.1%), costicocartilages (9/26, 34.6%) and peripheral joints (5/26, 19.2%).

**Fig. 1:**
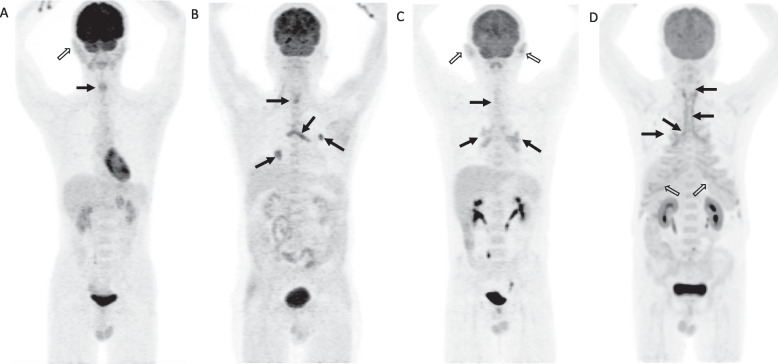
Representative cases of RP patients with airway involvement on PET of different patterns. **A** FDG-avid lesion was only seen in the larynx (focal pattern). **B** and **C** represented multifocal pattern that affected 2-3 segments of the airway. **B** Radiotracer uptake was identified mainly in the lobar and segmental bronchi: **C** Increased FDG uptake was seen in the bilateral main bronchi and extended to the left lower lobar bronchi. Focal uptake was also noted in the larynx and segmental bronchi. **D** Diffuse FDG uptake in the airway involving from the larynx extended to the lobar bronchi (diffuse pattern). Other involved sites (hollow arrows) included auricles (**A**, **B**) and costicartilages (**D**)

PET scans showed laryngo-tracheo-bronchial involvement in 94.2% (49/52) of patients. Laryngeal and tracheo-bronchial tree involvement was identified in 75.6 (37/49) and 89.8% (44/49) patients, respectively. The cricoid cartilage was the most frequently affected laryngeal cartilage (35/37, 94.6%), followed by arytenoid cartilage (30/37, 81.1%) and thyroid cartilages (27/37, 73.0%). However, the epiglottic cartilage was not affected in any of the patients. In terms of the tracheobronchial involvement, any segment could be positive on PET. The most commonly affected site was peripheral bronchi (39/52, 75.0%), followed by the main bronchi (34/52, 65.4%), and the trachea (29/52, 55.8%). Except 3 patients without any uptake in the whole airway, the other 49 patients were classified into focal, multifocal and diffuse uptake of 22.4% (11/49), 32.7% (16/49) and 44.9% (22/49) patients, respectively. Representative images were shown in Fig. 1 and Fig. S3-5 (Additional file 6). In patients with focal pattern, the larynx or peripheral airway was the most commonly affected site, while no patient exhibited sole main bronchi involvement. The median SUVmax of the laryngo-tracheo-bronchial tree was 3.5 [3.0-4.8], with4.0 [3.3-5.1] in the larynx and 3.5 [2.8-4.6] in the tracheo-bronchial tree. In addition, the TLG of the whole airway and peripheral airway in patients with previous corticosteroid therapy were significantly lower than those with corticosteroid therapy (both *p* < 0.05), although no similar difference was observed in the SUVmax. Nevertheless, there was no difference in the distribution of FDG-avid lesions between treated and non-treated patients (larynx: 76.9% vs. 53.8%, *p* = 0.159; tracheo-bronchial tree: 84.6% vs. 84.6%, *p* > 0.99). More data were shown in Table S3 (Additional file 7). Detailed quantitative PET parameters were shown in Table S4 (Additional file 8). Further comparison of clinical characteristics among the three groups was listed in Table S5 (Additional file 9). This result showed that patients with diffuse pattern demonstrated the highest level of inflammatory markers (ESR: *p* = 0.007; CRP: *p* = 0.002) compared with the others. In addition, the diffuse pattern was also associated with worse long-term outcomes (i.e. VAS score of symptoms).

In terms of CT features, 53.8% (28/52) patients showed structural abnormalities in the larynx, including cartilaginous destruction (13/28, 46.4%), soft-tissue swelling (24/28, 85.7%) and glottic/subglottic stenosis (16/28,57.1%). The most common sign of the tracheo-bronchial involvement was wall thickening with or without calcification (52/52, 100%). Among the 50 patients presenting with tracheal wall thickening, 70.0% (35/50) of them demonstrated posterior wall spared, while the other 30.0% (15/50) of patients revealed circumferential thickening (*p* = 0.377). Higher SUVmax and TLG of the trachea was found in patients with circumferential thickening for untreated patients (SUVmax: *p* = 0.024, TLG: *p* = 0.039), but not the case for patients undergoing corticosteroid treatment. Moreover, for patients with circumferential thickening, SUVmax and the TLG of the trachea demonstrated a decreased tendency in those with treatment (SUVmax: *p* = 0.043, TLG:* p* = 0.143), but not for patients sparing of posterior wall. Representative cases are shown in Fig. S6 (Additional file 10). Other CT findings included luminal stenosis (44/52, 84.6%), pulmonary inflammation presenting as ground-glass opacities (GGO), patchy consolidation with or without FDG uptake (28/52, 53.8%). These CT features were not significantly different between patients with treatment or not (Table S6, Additional file 11).

### Comparison of PET and bronchoscopy in the diagnosis of RP

The comparison of PET and bronchoscopy in lesion detection of the airway was shown in Table 2. Forty-three patients underwent bronchoscopy within 14 days of PET/CT. Positive findings of the trachea, main bronchi and lobar-segmental bronchi were shown in 39 (90.7%), 28 (65.1%) and 28 (65.1%) patients, respectively. The most common findings of the trachea included mucous abnormalities (32/43, 74.4%), tracheo-bronchial edema (23/43, 53.5%) and stenosis (20/43,46.5%). Tracheomalacia was identified in 34.9% (15/43) of patients. Abnormalities below the trachea could not be assessed in 3 patients, nor could be evaluated in 2 patients in the peripheral airways due to severe stenosis. Bronchoscopy showed a higher positive rate than PET in detecting tracheal abnormalities (90.7% vs.53.5%, *p* = 0.039) but a lower positive rate in the detection of lesions in the peripheral airways (65.1% vs. 79.1%, *p* = 0.046).

**Table 2:** Comparison of ^18^F-FDG PET and bronchoscopy in diagnosis of airway involvement in RP

	**Positive rate (*n* = 43)**		***P***
	**PET N (%)**	**Bronchoscopy N (%)**	
**Trachea**	23 (53.5)	39 (90.7)	***0.039***
**Main bronchi**	22 (51.2)	28 (65.1)	0.348
**Left**	19 (44.2)	26 (60.5)	> 0.99
**Right/bronchus intermediate**	20 (46.5)	24 (55.8)	0.542
**Lobar-segmental (peripheral) bronchi**	34 (79.1)	28 (65.1)	***0.046***
**Left**	32 (74.4)	25 (58.1)	0.156
**Right**	31 (72.1)	25 (58.1)	***0.014***

### Correlation of PET parameters with serological inflammatory markers and lung function

Serological inflammatory markers CRP and ESR were compared with PET parameters. Both SUVmax and TLG of the whole airway were positively correlated with CRP and ESR (all *p* < 0.05, Table 3). Furthermore, FDG uptake of the whole airway was higher in patients with elevated CRP levels compared with those in the normal range as was shown in Fig. S7 (Additional file 12), while the trend was not significant for ESR. Although the difference was not statistically significant, patients with increased CRP also showed a trend of FDG accumulation in ≥ 2 cartilages (*p* = 0.06).

**Table 3:** The correlation of serological markers and PET-based parameters of the airway

**Serological markers**	**Correlation with PET-based parameters of the airway**					
	**Whole airway-SUVmax [*rs* (*p*)]**	**Whole airway**-**TLG [*rs* (*p*)]**	**Central airway-SUVmax [*rs* (*p*)]**	**Central airway-TLG [*rs* (*p*)]**	**Peripheral airway-SUVmax [*rs* (*p*)]**	**Peripheral airway-TLG [*rs* (*p*)]**
**CRP**	0.418(0.007)	0.477(0.002)	0.325(0.041)	0.371(0.018)	0.482(0.002)	0.396(0.011)
**ESR**	0.408(0.006)	0.488(0.001)	0.252(0.099)	0.35(0.02)	0.413(0.005)	0.319(0.035)

*ESR* Erythrocyte sedimentation rate, *CRP* C-reactive protein, *SUVmax* Maximum standardized uptake value, *TLG* Total lesion glycolysis

**Fig. 2:**
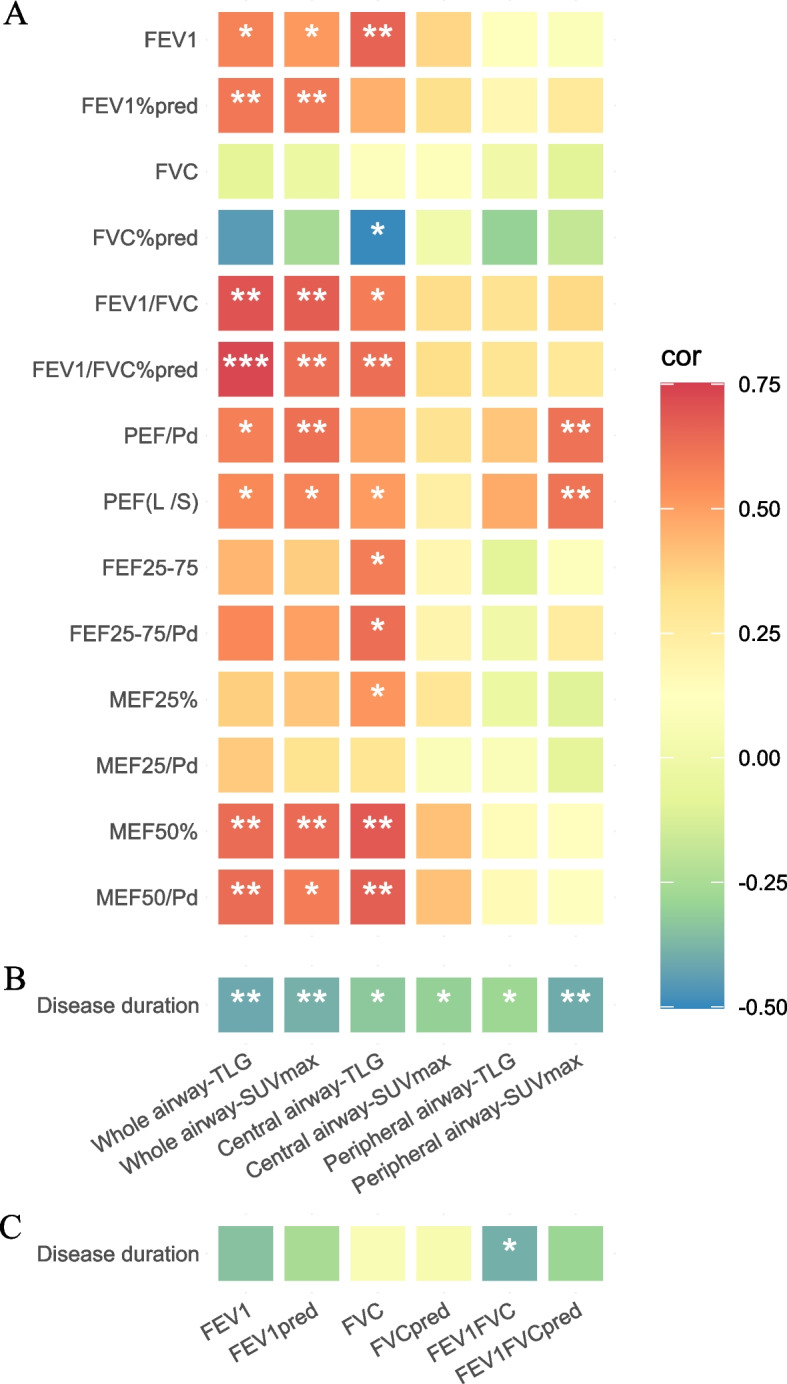
Positive correlation was shown between spirometric indicators and PET parameters (**A**), whereas negative correlation between disease duration and PET parameters (**B**)/spirometric indicators (**C**) were indicated

**Fig. 3:**
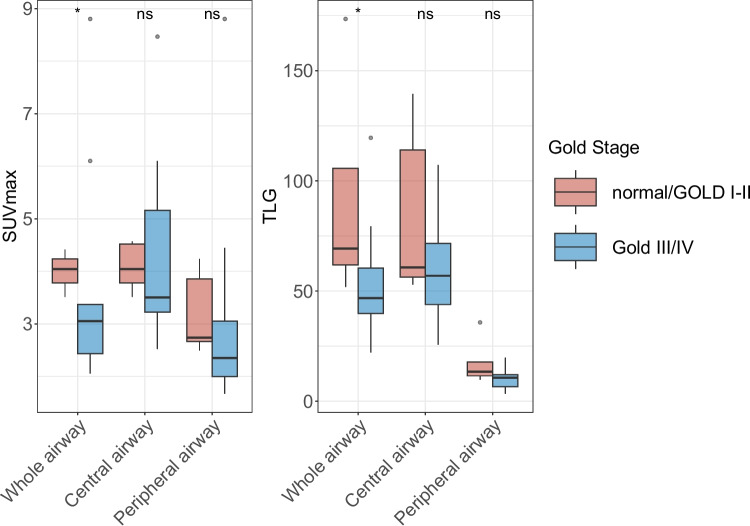
FDG activity of the airway was higher in patients with normal/GOLD I-II patients compared with GOLD III/IV stage

PET parameters were compared with PFTs results in 36 patients who underwent spirometry tests, while 18 of them completed post-bronchodilator (BD) testing. Figure 2A showed PET-derived parameters positively correlated with spirometric variables presented in both central and peripheral airways. The highest correlation coefficient results were observed between the TLG of the whole airway and the FEV1/FVC% pred (*rs* = 0.73, *p* < 0.001). Additionally, disease duration is negatively correlated with both PET parameters (Fig. 2B) or PFT indicators (Fig. 2C). Considering the degree of airway obstruction, SUVmax and TLG of the whole airway were lower in patients with higher stages (GOLD III/IV) (Fig. 3).

### Prognostic value of PET/CT in RP patients with airway involvement

**Fig.4:**
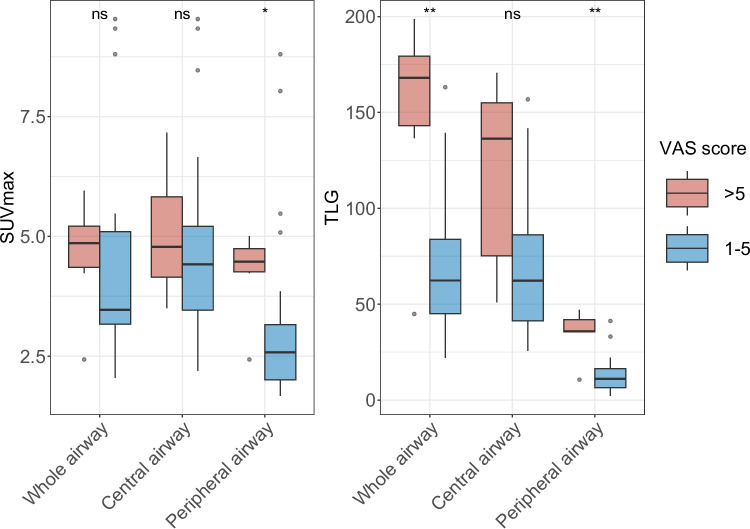
SUVmax and TLG of the airway in prediction of VAS score of RP patients. Patients with lower VAS of airway symptoms showed lower TLG of the whole airway and peripheral bronchi

Long-term follow-up was conducted for 41 RP patients with airway involvement, with a median follow-up of 5.8 years. Twenty-eight patients (68%) completed a questionnaire assessing respiratory symptoms, pulmonary function, subjective feeling and quality of life (QoL). Details were elicited in Table 4. In general, repetitive respiratory symptoms occurred in 75.0% (21/28) of patients. In terms of pulmonary function, mMRC with scores 1, 2 and 3-5 were seen in 32.1% (9/28), 50.0% (14/28) and 25.0% (7/28) of patients respectively. Patients with lower VAS of airway symptoms showed lower TLG of the whole airway and peripheral bronchi (Fig. 4). However, PET-based parameters showed no correlation with respiratory symptoms or mMRC scales. (Fig. S8, Additional file 13).

**Table 4:** Long-term outcome of RP patients

**Variables (total points)**	**Median (IQR, range) / N (%)**
**Respiratory symptoms (5**-**20)**	8 (6.5-11,5-16)
Neck/chest pain (4)	1 (1-1,1-4)
Hoarseness (4)	1 (1-1,1-4)
Cough (4)	2 (1-3,1-4)
Dyspnea (4)	2.5 (1-3,1-4)
Frequency (4)	2 (2-2.8,1-4)
**Dyspnea scale according to mMRC (1**-**5)**	2 (1-2,1-5)
Dyspnea with strenuous exercise (1)	9 (30%)
Dyspnea when hurrying or walking up a slight hill (2)	14 (46.7%)
Walks slower than people of the same age because of dyspnea or has to stop for breath when walking at own pace (3)	3 (10%)
Stops for breath after walking 100 yards (91 m) or after a few minutes (4)	2 (6.7%)
Too dyspneic to leave house or breathless when dressing (5)	2 (6.7%)
**VAS of airway symptoms (0**-**10)**	3 (2-5,1-10)
**QoL (3**-**6)**	
Good mental health (2)	24 (80.0%)
Good appetite (2)	27 (90.0%)
Well sleep (2)	25 (83.3%)
**Confidence about the disease (2**-**4)**	
Satisfied with current health condition (2)	21 (70.0%)
Have confidence in the future (2)	26 (86.7%)
**Total points (45)**	18 (15.5-25)

*mMRC* Modified Medical Research Council, *VAS* Visual analog scale, *QoL* Quality of life

Finally, 5 patients (5/41, 12.2%) died during the follow-up. Detailed information about the deaths was shown in Table S7 (Additional file 14). Two patients died from respiratory diseases with 1 due to RP, whereas 3 patients died due to malignancy, namely breast cancer (*n* = 1), lymphoma (*n* = 1) and laryngocarcinoma (*n* = 1).

## Discussion

This study comprehensively investigated the clinical and imaging features of RP with airway involvement, assessing the predictive value of PET/CT in diagnosis, disease severity, and prognosis. To our knowledge, this is the largest cohort of RP patients with airway involvement or undergoing PET/CT. The study presents several major findings: 1) PET demonstrated a favorable performance (positive rate: 94.2%) in detecting larynx/tracheobronchial lesions, clearly visualizing the extent of airway inflammation for both central and peripheral airways; 2) This study initially proposed three distinct patterns of airway involvement based on the number and distribution of FDG-avid segments; 3) PET parameters (i.e., SUVmax, TLG) significantly correlated with disease activity, pulmonary function, and long-term subjective respiratory symptoms in RP patients. These results may provide valuable new insights and evidence supporting the clinical application of PET/CT in patients with RP, particularly for those with airway involvement.

RP is a relatively rare systemic disorder characterized by diverse and nonspecific clinical presentations [5]. Airway involvement, though not uncommon, can lead to delays in diagnosis and poor outcomes, especially when respiratory symptoms present as the sole or initial manifestation [5, 6]. Inflammatory blood markers such as ESR and CRP may be useful for evaluating disease activity, but they are neither sensitive nor specificity [22]. Although chest CT is a routine modality for assessing thoracic disease, it cannot provide functional information. Therefore, there is a need to develop a reliable and objective approach to evaluate disease activity and severity.

Previous studies have suggested PET/CT as a valuable tool for assessing and monitoring RP, particularly in detecting airway involvement and establishing diagnosis for patients with respiratory symptoms [13, 16]. Similarly, the high positive rate of 94.2% in the airway lesions on PET was shown in our study, supporting its usefulness in the detection. Moreover, in those patients with respiratory symptoms as their only manifestation of RP, PET/CT may help to establish the diagnosis by showing localized FDG uptake in areas other than thoracic abnormalities, such as auricular, nasal cartilages, etc. This finding is consistent with previous studies [16, 23, 24]. These results indicate the notable strengths of PET/CT in early identification of tracheobronchial tree involvement and simultaneous detection of extra-thoracic lesions in patients with RP.

Several potential features of PET/CT imaging for RP have been proposed in previous studies [14–16], and the presence of symmetrical FDG uptake of cartilages appears to be the main pattern. In our study, PET findings of airway involvement were classified into three distinct patterns based on the distribution of involved segments: focal (1 site), multifocal (2-3 sites), and diffuse patterns (4 sites). The diffuse pattern was the most predominant form of airway involvement on PET, similar to previous typical imaging results [10, 23]. Our classification may suggest differences in airway involvement in development among various subtypes of RP, leading to an in-depth understanding the evolution of imaging findings in this rare disease. Furthermore, these PET patterns reflect inflammation extent/activity and long-term outcome to some extent, providing additional evidence for clinical management of RP (i.e., local or systemic intervention).

Another interesting finding is that 30.0% of our patients showed thickened posterior wall, which was considered not to be affected in RP due to lacking of cartilages [3, 9, 25]. The phenomenon was also noted in Lin’s research for 28.6% (2/7) of patients [26]. Our study explained the extent of thickened wall might be correlated with the degree of inflammation as was shown by ^18^F-FDG uptake of the trachea.

Bronchoscopy is a sensitive method for detecting cartilage lesions and abnormalities, as well as large airway stenosis, malacia, mucous abnormalities, etc. [23]. However, bronchoscopy may have limited sensitivity for the identification or diagnosis of early airway lesions in RP as well as in the detection of peripheral airway involvement, particularly in bronchial segments below the segmental bronchi. Furthermore, bronchoscopy is invasive and may consequently exacerbate mucosal swelling or cartilage inflammation via mechanical stimulation [27]. Our results revealed a complementary role of PET in this regard. PET may provide more objective and comprehensive insights into the extent and range of RP involvement. Moreover, PET can be helpful in guiding the biopsy site, even if in locations where abnormalities are not readily apparent, improving biopsy accuracy [23]. Therefore, the combination of bronchoscopy and PET/CT may be a more reliable and comprehensive approach for evaluating the extent of inflammation in RP.

As a common semiquantitative parameter in PET, SUVmax has been used for therapeutic evaluation of RP [11, 14, 16]. However, some patients experienced improved symptoms and reduced inflammation even when the SUVmax of specific sites on PET increased [6], suggesting SUVmax alone may not be sufficient. Moreover, previous studies failed to show a correlation between SUVmax and laboratory data due to the relatively small number of patients [14]. Our results provide the first evidence that both SUVmax and TLG of the whole airway positively correlated with whole-body inflammatory markers CRP and ESR. Patients with elevated CRP levels exhibited higher FDG uptake in the whole airway than those with normal levels. This suggests that for RP patients with airway involvement, FDG uptake of the airway may be one of the key sites for evaluating and monitoring the overall condition of RP. Furthermore, though TLG has been employed in some other inflammatory diseases (i.e. adult-onset Still’s disease, rheumatoid arthritis, IgG4-related disease, and large-vessel vasculitis) [28–31], our study first provides the positive correlation of TLG and inflammatory markers. In addition,our study revealed that TLG, but not SUVmax decreased after GC treatment, suggesting PET might offer a more comprehensive assessment of the inflammatory burden of the airway by adopting TLG. This approach combines information on both the extent and degree of inflammation, as opposed to solely relying on the airway's SUVmax.

Pulmonary function is essential for disease evaluation for RP. The most common abnormality is obstructive ventilation dysfunction, with FEV1, FVC, and FEV1/FVC indicating the severity of impairment of pulmonary function [9]. Our study showed the trend that lower FDG uptake of the airway corresponds to worse pulmonary function, and PET/CT might be helpful in assessing the severity of obstruction for RP patients who cannot tolerate PFT. One possible explanation might be due to the long disease duration of our cohort of patients who were possibly at an advanced stage of the disease; thus, lower FDG uptake might represent late pathological processes such as fibrosis rather than inflammation subsides, causing the positive correlation between FDG uptake and pulmonary function. The negative correlation between disease duration and some PFTs indicators also supported this hypothesis. The results need to be further validated with larger sample sizes.

Data about long-term outcomes of RP with airway involvement are scarce [5, 7, 32, 33]. Traditionally, it has been believed that RP patients with airway involvement have a poor prognosis [5], and there is a high incidence of RP-related morbidity requiring higher ICU occupancy [7]. Our study demonstrated RP-related death in only one (2.4%) patient which was lower than Hong et al. reported (3/12, 25%) [32], and tumor-related death in 3(7.3%) patients, indicating RP-related mortality is not so high, possibly due to the progress of interventional treatments. Our study also adopted VAS to assess RP and showed that patients who felt poor long-term subjective feeling of the airway symptoms assessed by VAS had higher TLG of the airway, indicating that early inflammatory burden may be correlated with long-term symptoms. In long-term management plans, PET results are worth considering. On the other hand, some patients still experience recurrent flare-ups or persistent chondritis-related symptoms, which remain a challenge. Therefore, it is essential to further optimize effective and sustainable treatment or management strategies for RP.

Although PET/CT has been attempted for the assessment of RP, there is still a lack of an optimal time-point for its application, which is crucial for clinical practice. Our study reveals that PET/CT may have diagnostic value for patients with airway involvement and extrathoracic lesions prior to steroid treatment. Notably, airway involvement is a potentially fatal type of RP, leading to airway narrowing, malacia, collapse and even death [7, 32]. In this setting, the early use of PET/CT may help to rapidly and accurately present those signs of heightened inflammation in airway cartilage. This provides a decision-making basis for invasive bronchoscopy for patients with suspected RP. Moreover, the FDG uptake-related parameters can also serve as a supplement to laboratory examinations and pulmonary function in the current absence of fully validated ideal indicators. In addition, PET appears to have potential value in treatment monitoring, but the optimal time interval for PET scanning after corticosteroid therapy remains unclear, with a reported range from 1 to 36 months [15, 34]. The cost-effectiveness and high radiation exposure associated with repeated PET scans should also be considered. Collectively, a standard strategy of PET/CT application in RP is necessary, and it is worth determining patients who will benefit most from PET/CT.

There are some limitations of the study. Firstly, this is a single-center study with a retrospective design, and potential selection bias may not be completely avoided. Secondly, the sample size of this study is relatively limited due to the rarity of the disease. Thirdly, the lack of follow-up PET/CT images did not allow for longitudinal observation of long-term changes in imaging features or further exploration its value in predicting treatment response. Despite these limitations, this study was performed with the largest cohort of RP with airway involvement, providing reliable evidence for the role of PET/CT in those patients.

## Conclusions

In conclusion, ^18^F-FDG PET/CT serves as a valuable tool for evaluating airway involvement in patients with RP. Besides extrathoracic lesions, PET/CT demonstrates high specificity in identifying airway lesions and comprehensively presents various types of distribution, which is advantageous for severity assessment and targeted biopsy. Quantitative PET parameters also offer evidence for monitoring disease activity, progression, and long-term outcomes. As such, PET/CT has the potential to be an ideal and objective approach for optimizing the management of RP with airway involvement, a persisting clinical challenge.

### Supplementary Information

**Additional file 1: Fig. S1.** (A-D) Bronchoscopy of RP patients showed that the mucosa of the trachea and main bronchus were severely swollen, accompanied by cartilage collapse and luminal stenosis.

**Additional file 2.** Survey questions for follow-up in RP patients.

**Additional file 3: Table S1.** Contents of PET/CT imaging analysis.

**Additional file 4: Fig.S2.** Example of 3D slicer software to calculate PET parameters. Region of interests (ROIs) of the airway were drawn on the CT images of PET/CT. Then these ROIs were projected to PET images, and PET paremeters (SUVmax, TLG etc.) were calculated automatically by software.

**Additional file 5: Table S2.** Regimes of previous treatment.

**Additional file 6: Fig. S3.** Two patients in focal pattern. (A,B) showed only laryngeal involvement; (C,D) showed bilateral lobar-segmental involvement. **Fig. S4.** Patient with multifocal pattern. Larynx, bilateral main bronchus and bilateral lobar-segmental involvement were demonstrated. **Fig. S5.** One patient with diffuse pattern. All four segments of the laryngo-tracheabronchial tree were involved.

**Additional file 7: Table S3.** Distribution of FDG-avid involvement on laryngo-tracheo-bronchial tree.

**Additional file 8: Table S4.** PET parameters of the airway.

**Additional file 9: Table S5.** Clinical features of patients with different imaging patterns.

**Additional file 10: Fig. S6.** Correlation of ^18^F-FDG uptake with different types of tracheal wall thickening.

**Additional file 11: Table S6.** CT features of the airway involvement on PET/CT.

**Additional file 12: Fig. S7.** Comparison of FDG uptake in patients with different CRP levels. Both SUVmax and TLG of the whole airway was higher in patients with elevated CRP level compared with those within normal range.

**Additional file 13: Fig. S8.** PET-based parameters showed no correlation with respiratory symptoms (A1-2) or mMRC scales (B1-2).

**Additional file 14: Table S7.** Information of the 5 dead patients.

## Data Availability

The datasets generated and/or analysed during the current study are not publicly available due to another related unpublished paper but are available from the corresponding author on reasonable request.

## Abbreviations

PET/CT: Positron emission tomography/computed tomography
FDG: Fluorodeoxyglucose
RP: Relapsing polychondritis
SUVmax: Maximum standard uptake value
TLG: Total lesion glycolysis
PFT: Pulmonary function test
CRP: C-reactive protein
ESR: Erythrocyte sedimentation rate
^18^F-FDG: Fluorine-18 fludeoxyglucose
DICOM: Digital Imaging and Communications in Medicine
ATS/ESR: American Thoracic Society/European Respiratory Society;
GOLD: Global Initiative for Chronic Obstructive Lung Disease
mMRC: modified Medical Research Council
SGRQ: St George’s Respiratory Questionnaire
VAS: Visual Analogue Scale
WHO: World Health Organization
QoL: Quality of Life
