# Supplementary material for: Imaging features and clinical value of 18F-FDG PET/CT for predicting airway involvement in patients with relapsing polychondritis
Source: Arthritis Res Ther. 2023 Oct 14;25:198. doi: 10.1186/s13075-023-03156-x (PMC10576346; doi:10.1186/s13075-023-03156-x)
Supplement: Supplementary file 1 — Additional file 1: Fig. S1. (A-D) Bronchoscopy of RP patients showed that the mucosa of the trachea and main bronchus were severely swollen, accompanied by cartilage collapse and luminal stenosis. [file 13075_2023_3156_MOESM1_ESM.pdf]

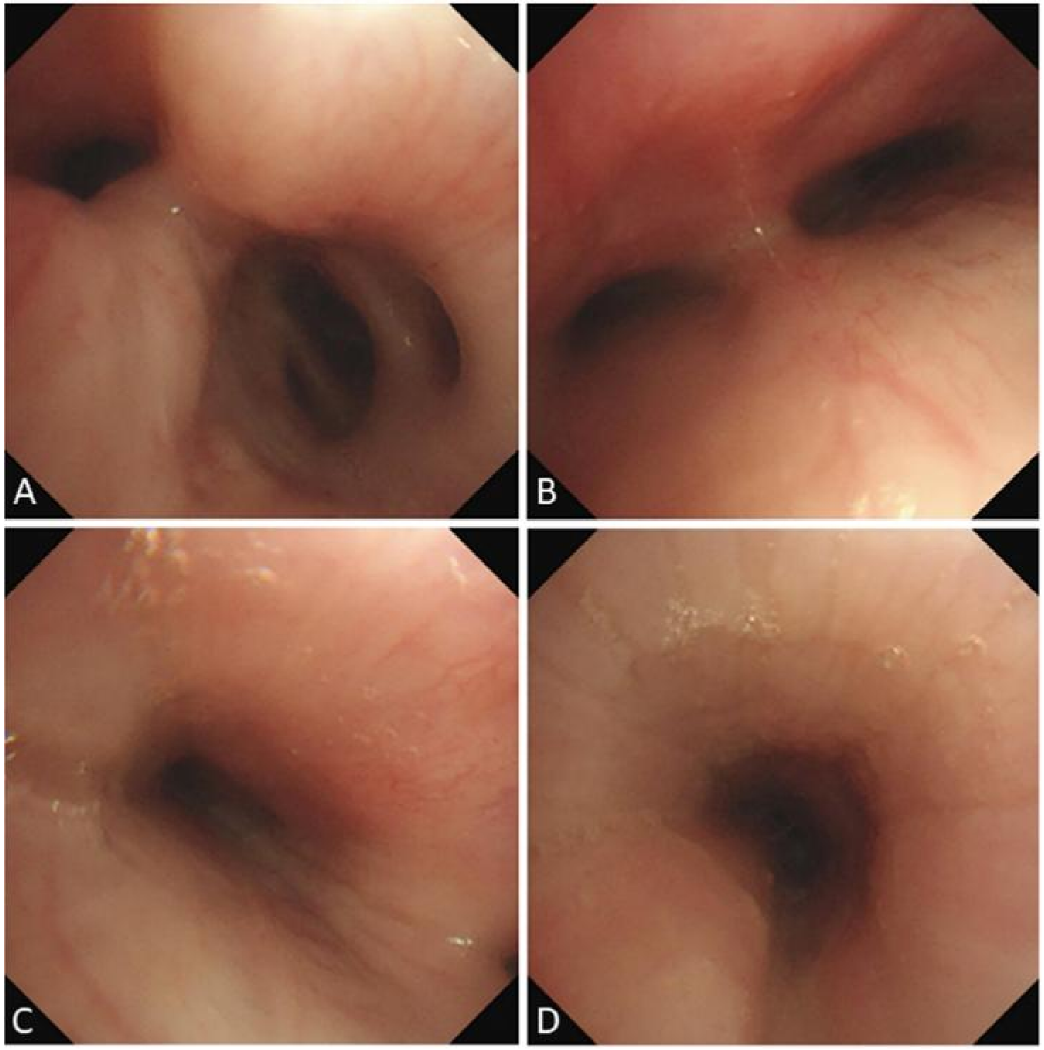

Fig.S1 (A-D) Bronchoscopy of RP patients showed that the mucosa of the trachea and main bronchus were severely swollen, accompanied by cartilage collapse and luminal stenosis.
