# Supplementary material for: Imaging features and clinical value of 18F-FDG PET/CT for predicting airway involvement in patients with relapsing polychondritis
Source: Arthritis Res Ther. 2023 Oct 14;25:198. doi: 10.1186/s13075-023-03156-x (PMC10576346; doi:10.1186/s13075-023-03156-x)
Supplement: Supplementary file 2 — Additional file 2. Survey questions for follow-up in RP patients. [file 13075_2023_3156_MOESM2_ESM.docx]

**Additional file 2. Survey questions for follow-up in RP patients**

**Part 1 respiratory symptoms**

Have you experienced any of those symptoms below in the past year?

(A：1 point; B: 2 points; C: 3 points; D: 4 points)

1. Neck pain or chest pain
2. Hoarseness
3. Cough
4. Dyspnea
5. Never.
6. Yes, but subside on their own.
7. Yes and subside after take medicine.
8. Yes and require hospitalization.
9. The frequency of the symptom
10. Never
11. <twice/year

C. >twice/year

D. Constantly

**Part 2 related to pulmonary function**

6. mMRC Dyspnea Scale

1. . Dyspnea with strenuous exercise (1)
2. . Dyspnea when hurrying or walking up a slight hill (2)

(3) Walks slower than people of the same age because of dyspnea or has to stop for breath when walking at own pace (3)

(4). Stops for breath after walking 100 yards (91 m) or after a few minutes (4)

(5). Too dyspneic to leave house or breathless when dressing (5)

1. Compared with the worst time, how would you rate yourself now? (0-10 points, 0 represent the best and 10 means at the same level of the worst)

**Part 3 Quality of life (Y=1 point; N=2 points)**

8. Are you satisfied with your mental health? Y/N

9. Do you have good appetite? Y/N

10. Are you satisfied with your sleep? Y/N

**Part 4 Confidence about the disease (Y=1 point; N=2 points)**

1. Are you satisfied with current health condition? Y/N
2. Do you have confidence in the future in regard to the disease? Y/N

Thanks for your attention!
