# Supplementary material for: Imaging features and clinical value of 18F-FDG PET/CT for predicting airway involvement in patients with relapsing polychondritis
Source: Arthritis Res Ther. 2023 Oct 14;25:198. doi: 10.1186/s13075-023-03156-x (PMC10576346; doi:10.1186/s13075-023-03156-x)
Supplement: Supplementary file 3 — Additional file 3: Table S1. Contents of PET/CT imaging analysis. [file 13075_2023_3156_MOESM3_ESM.docx]

**Table S1. Contents of PET/CT imaging analysis**

| **Items** | **Definition** |
| --- | --- |
| **PET items** |  |
| Biological uptake in larynx | Symmetrical visual F-18 FDG uptake in the laryngeal muscles or vocal cords |
|  |  |
| Abnormal uptake in larynx | Uptake in the larynx other than biological uptake |
|  |  |
| Abnormal tracer accumulation in the tracheo-bronchial tree | 1. Uptake in the airway wall corresponding to CT 2. Uptake higher than the surrounding background 3. The distribution of FDG uptake was consistent with the shape of the tracheo-bronchial tree |
| **CT items** |  |
| Airway wall thickening | Thickness of the involved segments of the trachea or main bronchi greater than 2 mm |
|  |  |
| Airway stenosis | Luminal width smaller than 25% of the other part of the airway |
