# Supplementary material for: Imaging features and clinical value of 18F-FDG PET/CT for predicting airway involvement in patients with relapsing polychondritis
Source: Arthritis Res Ther. 2023 Oct 14;25:198. doi: 10.1186/s13075-023-03156-x (PMC10576346; doi:10.1186/s13075-023-03156-x)
Supplement: Supplementary file 4 — Additional file 4: Fig.S2. Example of 3D slicer software to calculate PET parameters. Region of interests (ROIs) of the airway were drawn on the CT images of PET/CT. Then these ROIs were projected to PET images, and PET paremeters (SUVmax, TLG etc.) were calculated automatically by software. [file 13075_2023_3156_MOESM4_ESM.pdf]

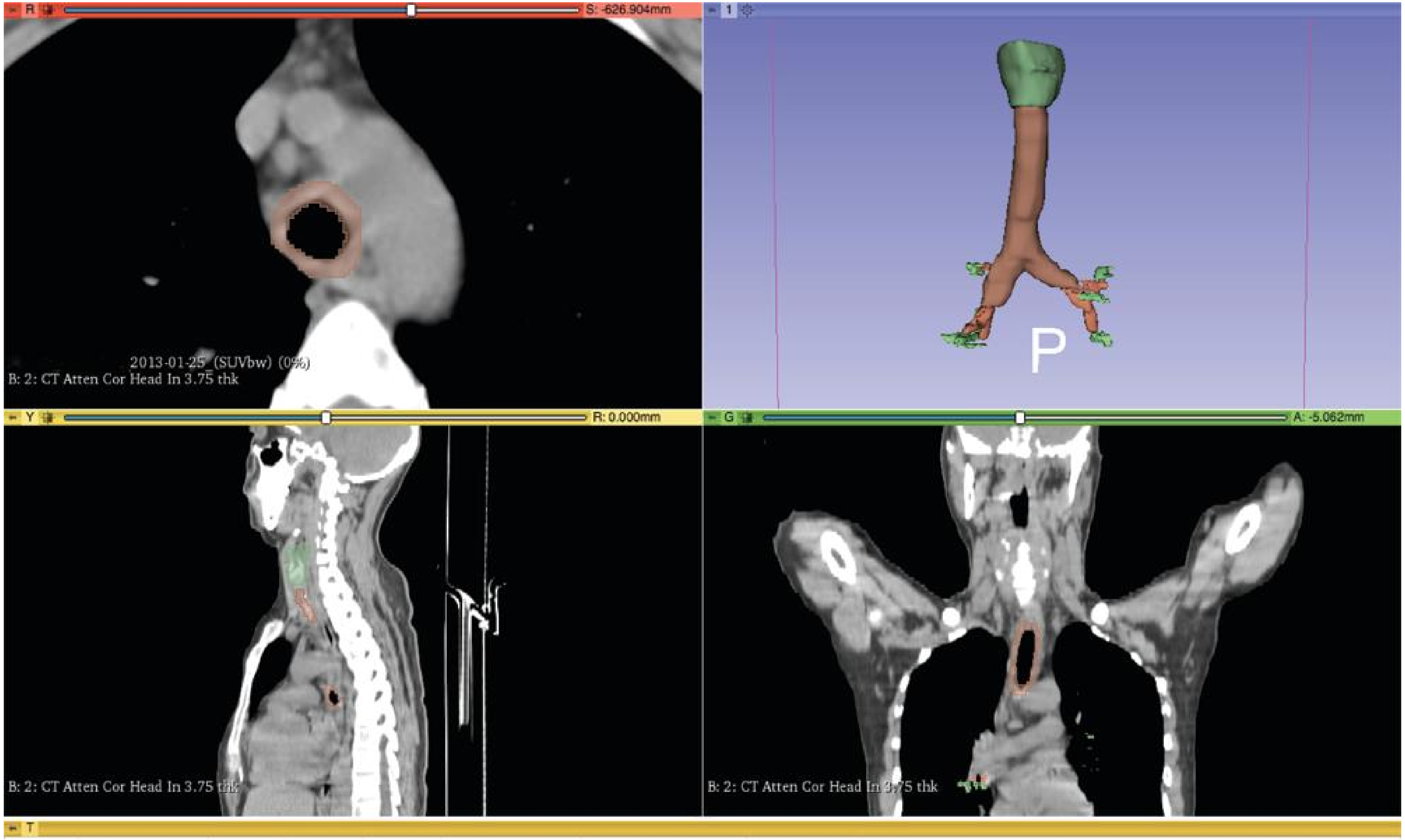

Fig.S2 Example of 3D slicer software to calculate PET parameters. Region of interests (ROIs) of the airway were drawn on the CT images of PET/CT. Then these ROIs were projected to PET images, and PET paremeters (SUVmax, TLG etc.) were calculated automatically by software.
