# Supplementary material for: Imaging features and clinical value of 18F-FDG PET/CT for predicting airway involvement in patients with relapsing polychondritis
Source: Arthritis Res Ther. 2023 Oct 14;25:198. doi: 10.1186/s13075-023-03156-x (PMC10576346; doi:10.1186/s13075-023-03156-x)
Supplement: Supplementary file 5 — Additional file 5: Table S2. Regimes of previous treatment. [file 13075_2023_3156_MOESM5_ESM.docx]

**Table S2. Regimes of previous treatment**

| **Regimes of previous treatment** | **Value (median[IQR])** |
| --- | --- |
| **Steroid treatment** | 13 |
| Highest dosage (mg/d) | 40 [27.5, 40] |
| Duration (d) | 158 [119.5-987.5] |
| **Immunosuppressive drugs** | 4 |
| Cyclophosphamide | 1 |
| Cumulative dose (g) | 4.2 |
| Duration (d) | 170 |
| Methotrexate | 3 |
| Dosage (mg/w) | 12.5 [10,12.5] |
| Duration (d) | 180 [84, 180] |
