# Supplementary material for: Imaging features and clinical value of 18F-FDG PET/CT for predicting airway involvement in patients with relapsing polychondritis
Source: Arthritis Res Ther. 2023 Oct 14;25:198. doi: 10.1186/s13075-023-03156-x (PMC10576346; doi:10.1186/s13075-023-03156-x)
Supplement: Supplementary file 6 — Additional file 6: Fig. S3. Two patients in focal pattern. (A,B) showed only laryngeal involvement; (C,D) showed bilateral lobar-segmental involvement. Fig. S4. Patient with multifocal pattern. Larynx, bilateral main bronchus and bilateral lobar-segmental involvement were demonstrated. Fig. S5. One patient with diffuse pattern. All four segments of the laryngo-tracheabronchial tree were involved. [file 13075_2023_3156_MOESM6_ESM.pdf]

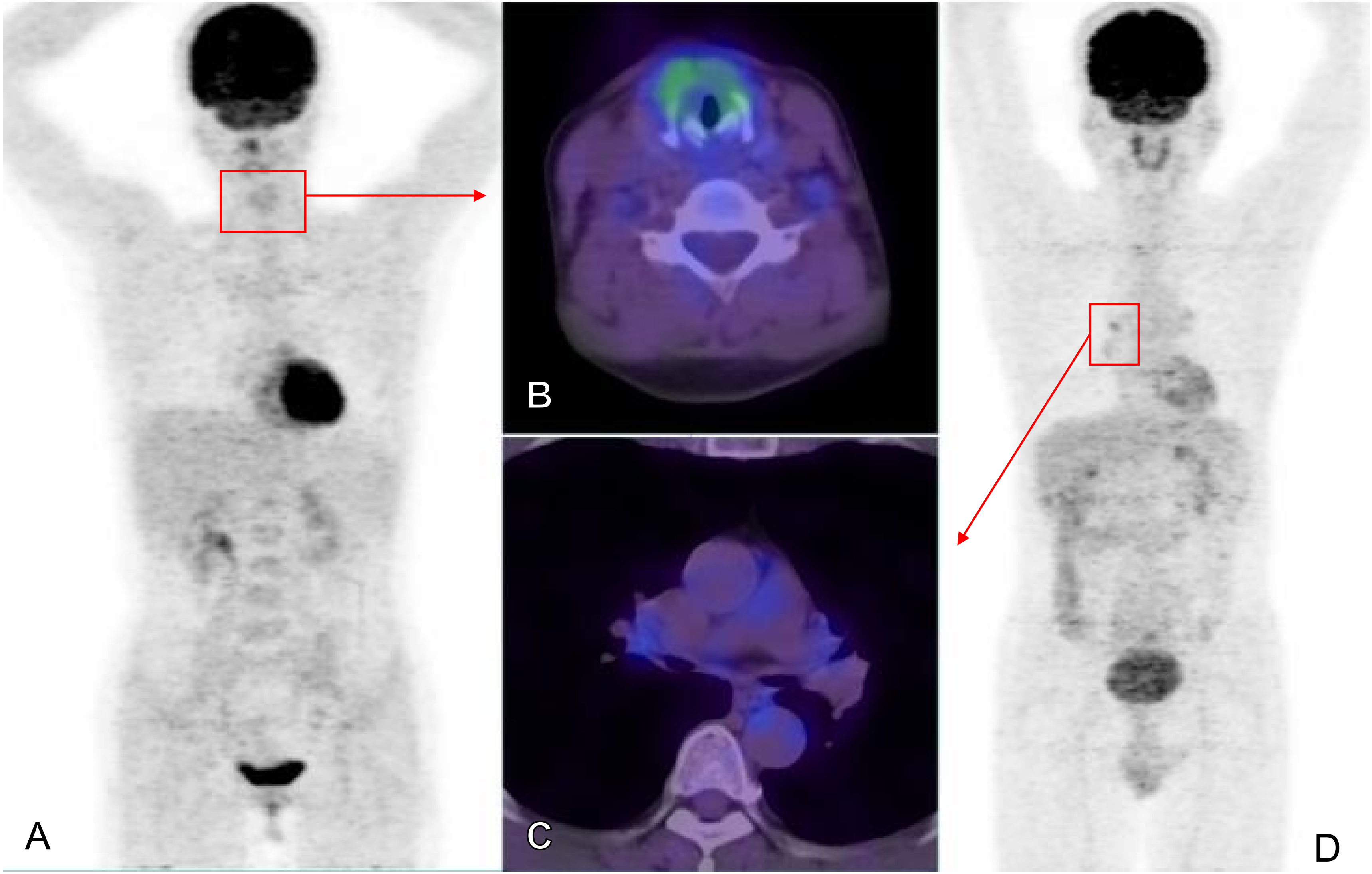

Fig.S3 Two patients in focal pattern. (A,B) showed only laryngeal involvement; (C,D) showed bilateral lobar-segmental involvement.

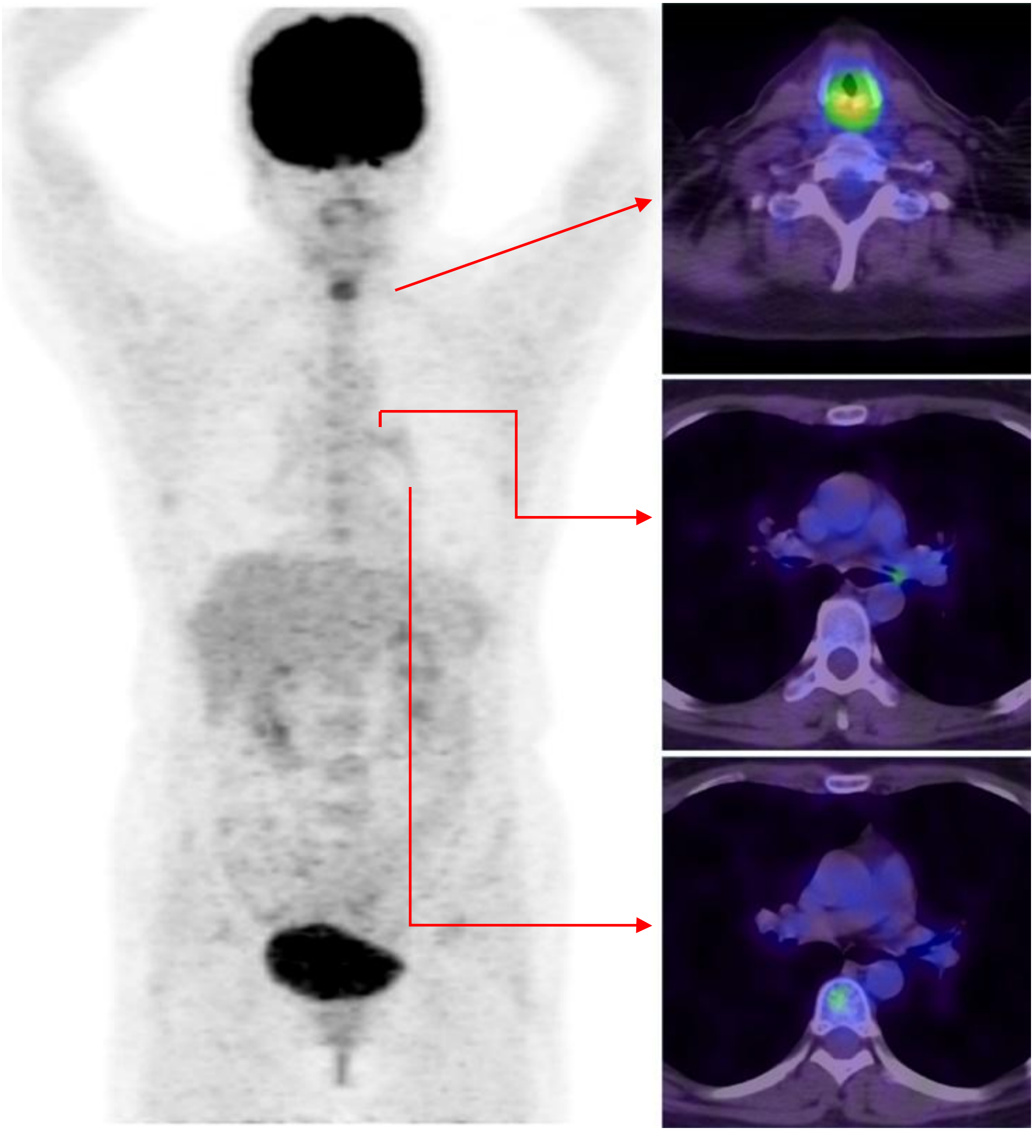

Fig. S4 Patient with multifocal pattern. Larynx, bilateral main bronchus and bilateral lobar-segmental involvement were demonstrated.

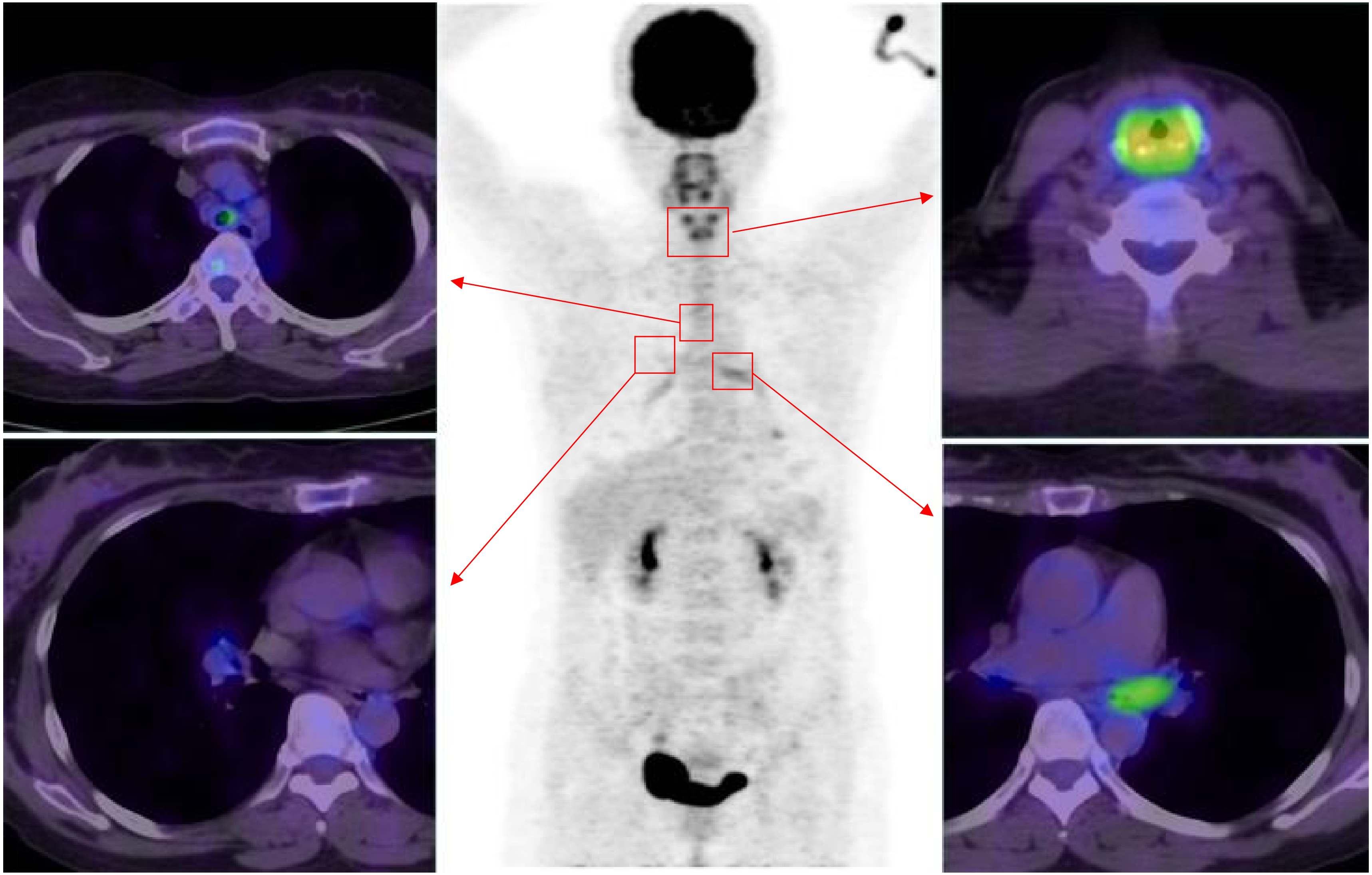

Fig. S5 One patient with diffuse pattern. All four segments of the laryngo-trachea-bronchial tree were involved.
