# Supplementary material for: Imaging features and clinical value of 18F-FDG PET/CT for predicting airway involvement in patients with relapsing polychondritis
Source: Arthritis Res Ther. 2023 Oct 14;25:198. doi: 10.1186/s13075-023-03156-x (PMC10576346; doi:10.1186/s13075-023-03156-x)
Supplement: Supplementary file 7 — Additional file 7: Table S3. Distribution of FDG-avid involvement on laryngo-tracheo-bronchial tree. [file 13075_2023_3156_MOESM7_ESM.docx]

**Table S3. Distribution of FDG-avid involvement on laryngo-tracheo-bronchial tree**

|  | **Untreated**  **(n= 39)** | **Treated**  **(n= 13)** | ***p***  **value** |
| --- | --- | --- | --- |
| **Larynx** | 30 (76.9%) | 7 (53.8%) | 0.159 |
| Cricoid cartilage | 28 (71.8%) | 7 (53.8%) | 0.309 |
| Arytenoid cartilage | 24 (61.5%) | 6 (46.2%) | 0.353 |
| Thyroid cartilages | 22 (56.4%) | 5 (38.5%) | 0.055 |
| **Tracheo-bronchial tree** | 33 (84.6%) | 11 (84.6%) | >0.99 |
| Trachea | 22 (56.4%) | 7 (53.8%) | >0.99 |
| Main bronchi and intermediate bronchus | 25 (64.1%) | 9 (69.2%) | >0.99 |
| Lobar bronchi | 27 (69.2%) | 10 (76.9%) | 0.732 |
| Segmental bronchi | 22 (56.4%) | 9 (69.2%) | 0.523 |
