# Supplementary material for: Imaging features and clinical value of 18F-FDG PET/CT for predicting airway involvement in patients with relapsing polychondritis
Source: Arthritis Res Ther. 2023 Oct 14;25:198. doi: 10.1186/s13075-023-03156-x (PMC10576346; doi:10.1186/s13075-023-03156-x)
Supplement: Supplementary file 8 — Additional file 8: Table S4. PET parameters of the airway. [file 13075_2023_3156_MOESM8_ESM.docx]

**Table S4 PET parameters of the airway**

|  | **Total (n=52)** | **Untreated patients**  **(n=39)** | **Treated patients**  **(n=13)** | ***p* value** |
| --- | --- | --- | --- | --- |
| Whole airways -SUVmax | 3.5 (3-4.8) | 4 (3.2-5) | 3.2 (2.7-3.9) | 0.115 |
| Whole airways -TLG | 64.3 (44.6-104) | 73.6 (46.1-115.4) | 47.6 (39.5-78.3) | **0.046** |
| Larynx-SUVmax | 4 (3.3-5.1) | 4 (3.3-5.1) | 3.9 (2.7-5.2) | 0.941 |
| Larynx-TLG | 25.8 (17.4-36.5) | 26.6 (17.4-39) | 20.3 (17.9-26.7) | 0.466 |
| Tracheobronchial tree-SUVmax | 3.5 (2.8-4.6) | 3.8 (3.2-4.7) | 3.2 (2.7-3.9) | 0.194 |
| Tracheobronchial tree-TLG | 39.5 (25.2-67.4) | 40.7 (27.7-79.8) | 28.2 (23.5-65.8) | 0.154 |
| Central airways -SUVmax | 4.2 (3.5-5.2) | 4.5 (3.5-5.2) | 3.9 (3.2-5.2) | 0.65 |
| Central airways -TLG | 63.6 (43.2-105.2) | 70.3 (49.9-107.1) | 51 (42-86.1) | 0.267 |
| Peripheral airways -SUVmax | 2.7 (2.2-4.1) | 3 (2.3-4.3) | 2.4 (1.8-2.7) | 0.101 |
| Peripheral airways -TLG | 11.4 (7.8-18.9) | 13.5 (8.4-20.1) | 9.1 (4.8-10.7) | **0.012** |

None of the data conform to the normal distribution, the p-value is calculated by the Kruskal-Wallis test.

Data were presented as Median(IQR).

SUVmax: maximal standardized uptake value; TLG: total lesion glycolysis.
