# Supplementary material for: Imaging features and clinical value of 18F-FDG PET/CT for predicting airway involvement in patients with relapsing polychondritis
Source: Arthritis Res Ther. 2023 Oct 14;25:198. doi: 10.1186/s13075-023-03156-x (PMC10576346; doi:10.1186/s13075-023-03156-x)
Supplement: Supplementary file 9 — Additional file 9: Table S5. Clinical features of patients with different imaging patterns. [file 13075_2023_3156_MOESM9_ESM.docx]

**Table S5. Clinical features of patients with different imaging patterns**

| **Characteristics** | **Focal pattern** | **Multifocal pattern** | **Diffuse pattern** | ***p* value** |
| --- | --- | --- | --- | --- |
| **Number of patients** | 11 | 16 | 22 |  |
| **Age (year)** | 51 [45-55] | 48 [35-54.25] | 44.5 [38.75-50] | 0.746 |
| **Course of disease (month)^#^** | 12 [4.5-18] | 12 [9-36] | 4 [3-7.5] | 0.0766 |
| **Sex** |  |  |  | 0.1428 |
| Male | 8 | 7 | 16 |  |
| Female | 3 | 9 | 6 |  |
| **Number of symptoms** |  |  |  | 0.3392 |
| 1-3 | 3 | 5 | 11 |  |
| >3 | 8 | 11 | 11 |  |
| **Patients** **previously treated with corticosteroid** | 3 | 4 | 5 | 0.9583 |
| **Inflammatory markers** |  |  |  |  |
| CRP (mg/L) | 0.82 [0.42-2.16] | 0.52 [0.17-1.35] | 8.75 [3.51-14.86] | 0.00743^*^ |
| ESR (mm/h) | 18.5 [15.25-45.25] | 33 [22-56] | 90 [38-120] | 0.00227^*^ |
| **Outcomes** |  |  |  |  |
| VAS | 6 | 11 | 13 | 0.0330^*^ |
| >4 | 0 | 2 | 7 |  |
| <=4 | 6 | 9 | 6 |  |
| mMRC | 6 | 11 | 13 | 0.313 |
| >3 | 0 | 3 | 4 |  |
| 1-3 | 6 | 8 | 9 |  |
| Total score of respiratory symptoms | 8 | 11 | 14 | 0.6956 |
| >8 | 2 | 6 | 7 |  |
| 1-8 | 6 | 5 | 7 |  |

Data were presented as Median[IQR].

# from the beginning of the symptoms to the day of diagnosis of RP

ESR, erythrocyte sedimentation rate; CRP, C-reactive protein; VAS, Visual Analogue Scale; mMRC, modified Medical Research Council
