# Supplementary material for: Imaging features and clinical value of 18F-FDG PET/CT for predicting airway involvement in patients with relapsing polychondritis
Source: Arthritis Res Ther. 2023 Oct 14;25:198. doi: 10.1186/s13075-023-03156-x (PMC10576346; doi:10.1186/s13075-023-03156-x)
Supplement: Supplementary file 10 — Additional file 10: Fig. S6. Correlation of 18F-FDG uptake with different types of tracheal wall thickening. [file 13075_2023_3156_MOESM10_ESM.pdf]

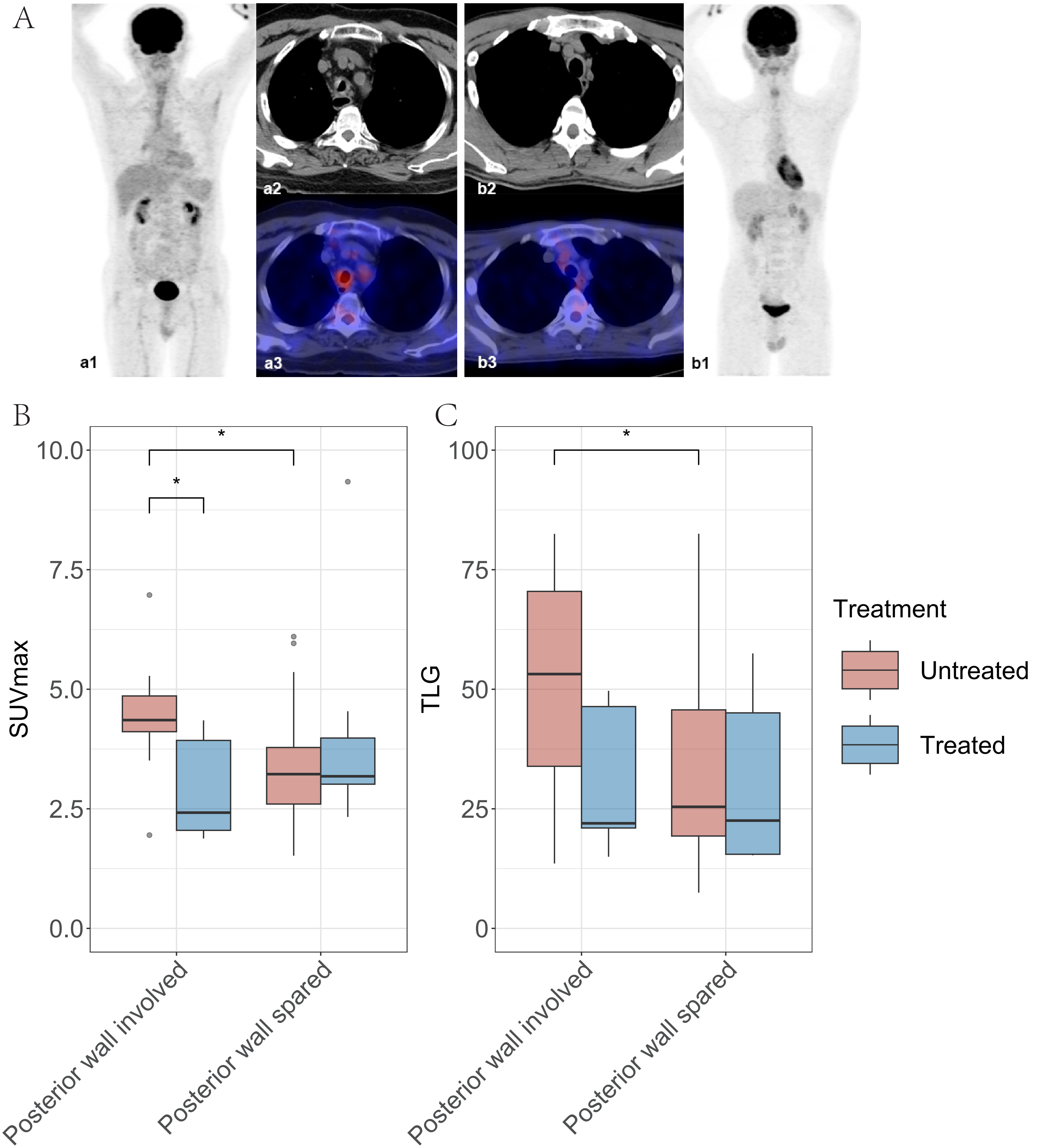

Fig.S6 Correlation of 18F-FDG uptake with different types of tracheal wall thickening. (A) Ring-like increased FDG uptake was seen in the thickened tracheal wall with posterior wall involved (a1-3) and (b1-3) showed another patient with thickening of the anterior and lateral walls of the trachea, the FDG uptake was not definite. (B) and (C) showed the SUVmax and TLG of the trachea were higher in untreated patients with circumferential thickened wall. Furthermore, the uptake decreased after therapy in patients with posterior wall involved. (*, $p < 0.05$)
