# Supplementary material for: Imaging features and clinical value of 18F-FDG PET/CT for predicting airway involvement in patients with relapsing polychondritis
Source: Arthritis Res Ther. 2023 Oct 14;25:198. doi: 10.1186/s13075-023-03156-x (PMC10576346; doi:10.1186/s13075-023-03156-x)
Supplement: Supplementary file 11 — Additional file 11: Table S6. CT features of the airway involvement on PET/CT. [file 13075_2023_3156_MOESM11_ESM.docx]

**Table S6. CT features of the airway involvement on PET/CT**

| **Imaging features** | **Untreated patients**  **(n = 39)** | **Treated patients**  **(n = 13)** | ***p* value** |
| --- | --- | --- | --- |
| **Larynx (n = 28)** |  |  |  |
| Cartilageous destruction (n = 13) | 8 (31.3%) | 5 (66.7%) | 0.125 |
| Soft tissue swelling, calcification (n = 24) | 19 (87.5%) | 5 (83.3%) | >0.99 |
| Glottic/Subglottic stenosis (n = 16) | 12 (68.8%) | 4 (41.7%) | 0.250 |
| **Tracheo-bronchial tree** |  |  |  |
| Wall thickening w/wo calcification (n = 52) | 39 (100%) | 13 (100%) | >0.99 |
| Circumferential thickening of the trachea (n = 15) | 10 (23.3%) | 5 (36.4%) | 0.483 |
| Luminal stenosis (n = 44) | 31 (76.7%) | 13 (95.5%) | 0.178 |
| Pulmonary inflammation (n = 28) | 17 (43.3%) | 11 (68.2%) | 0.096 |
