# Supplementary material for: Imaging features and clinical value of 18F-FDG PET/CT for predicting airway involvement in patients with relapsing polychondritis
Source: Arthritis Res Ther. 2023 Oct 14;25:198. doi: 10.1186/s13075-023-03156-x (PMC10576346; doi:10.1186/s13075-023-03156-x)
Supplement: Supplementary file 12 — Additional file 12: Fig. S7. Comparison of FDG uptake in patients with different CRP levels. Both SUVmax and TLG of the whole airway was higher in patients with elevated CRP level compared with those within normal range. [file 13075_2023_3156_MOESM12_ESM.pdf]

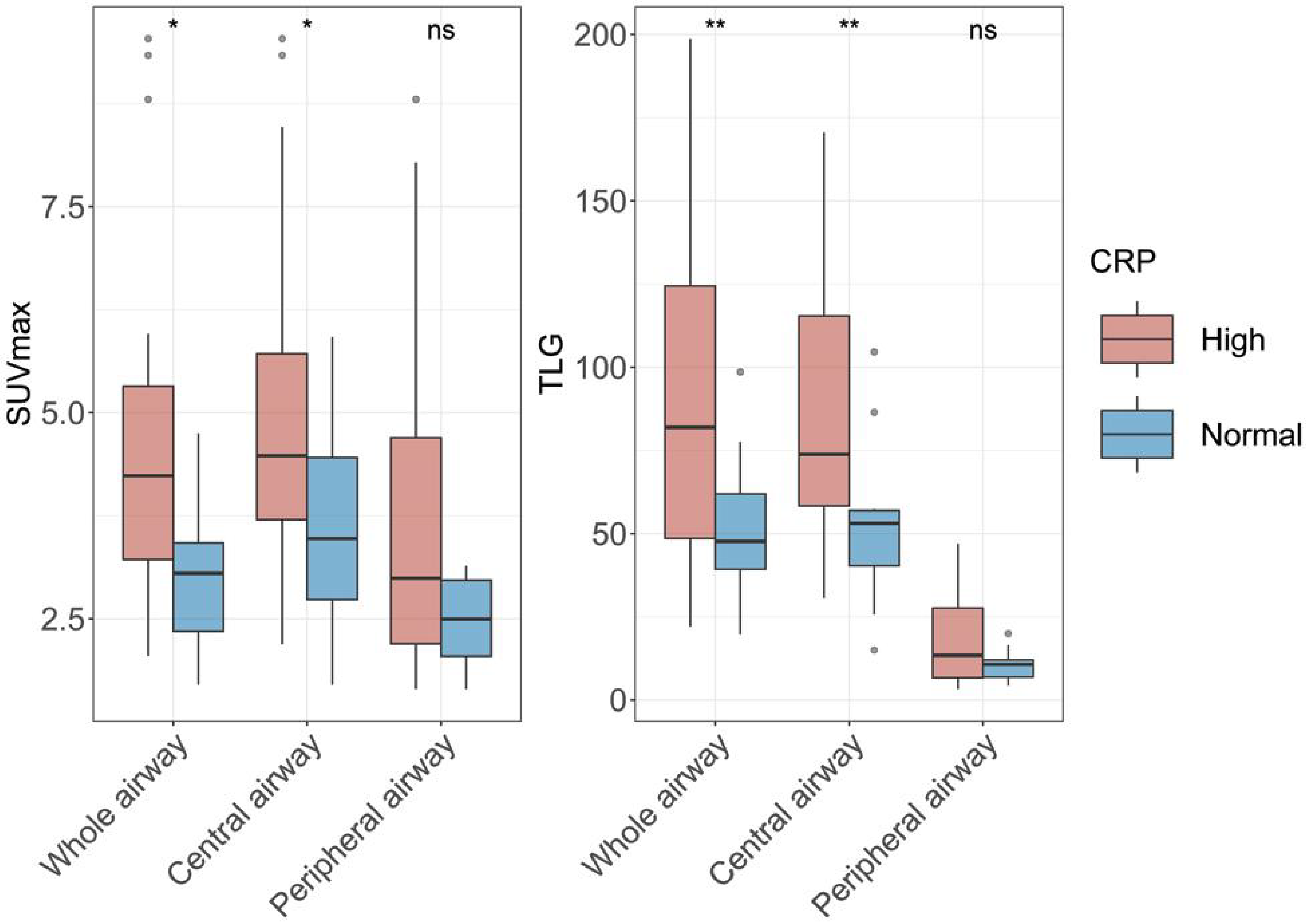

Fig.S7 Comparison of FDG uptake in patients with different CRP levels. Both SUVmax and TLG of the whole airway was higher in patients with elevated CRP level compared with those within normal range.
