# Supplementary figures and images for: Imaging features and clinical value of 18F-FDG PET/CT for predicting airway involvement in patients with relapsing polychondritis
Source: Arthritis Res Ther. 2023 Oct 14;25:198. doi: 10.1186/s13075-023-03156-x (PMC10576346; doi:10.1186/s13075-023-03156-x)

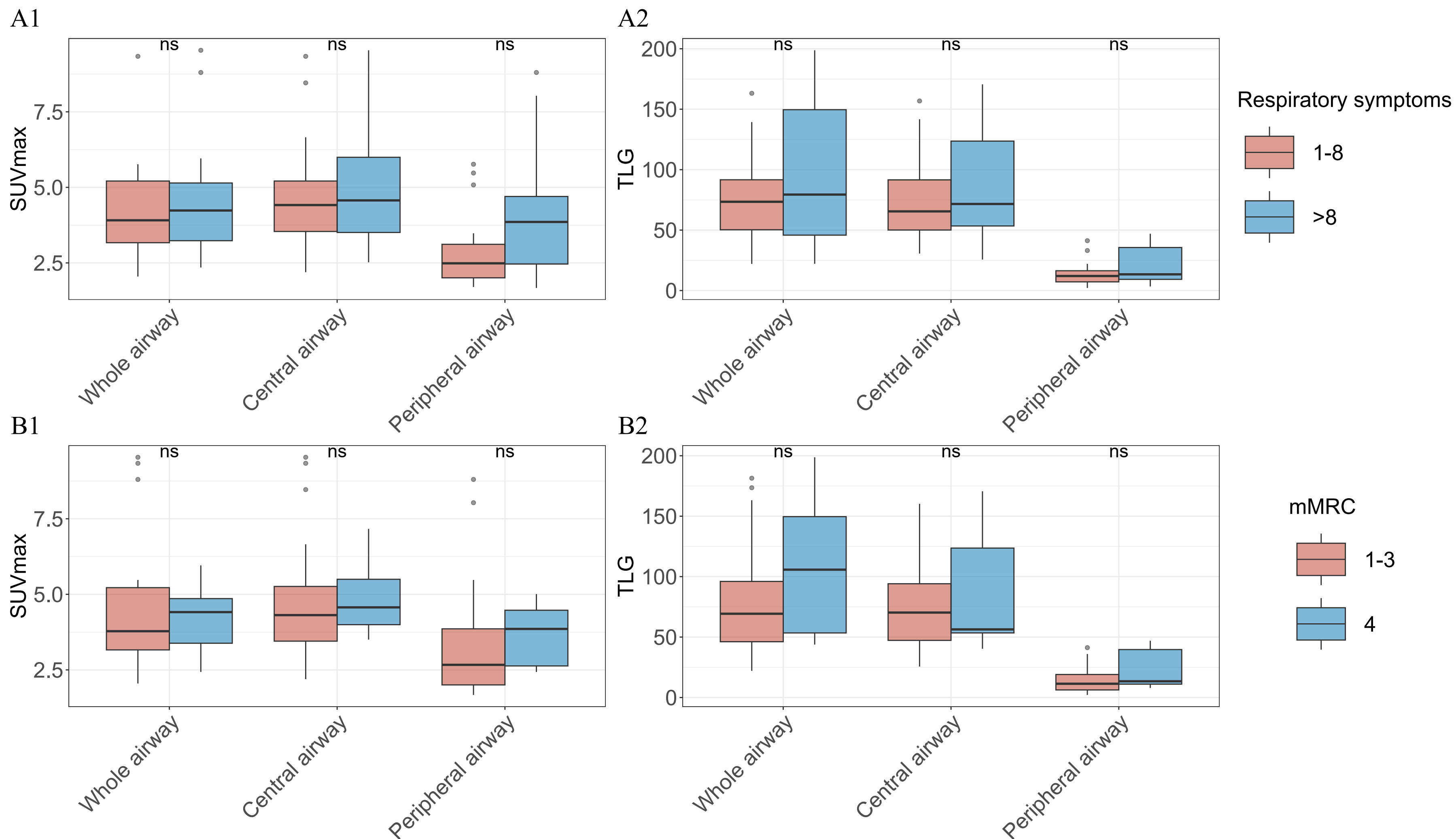

Fig.S8 PET-based parameters showed no correlation with respiratory symptoms (A1-2) or mMRC scales (B1-2).

Supplement: Supplementary file 13 — Additional file 13: Fig. S8. PET-based parameters showed no correlation with respiratory symptoms (A1-2) or mMRC scales (B1-2). [file 13075_2023_3156_MOESM13_ESM.pdf]
