# Supplementary material for: Imaging features and clinical value of 18F-FDG PET/CT for predicting airway involvement in patients with relapsing polychondritis
Source: Arthritis Res Ther. 2023 Oct 14;25:198. doi: 10.1186/s13075-023-03156-x (PMC10576346; doi:10.1186/s13075-023-03156-x)
Supplement: Supplementary file 14 — Additional file 14: Table S7. Information of the 5 dead patients. [file 13075_2023_3156_MOESM14_ESM.docx]

**Table S7. Information of the 5 dead patients**

| **Patient**  **No./Sex/Age** | **Following time (month)** | **PET/CT features of the airway** | | | | **Other features on PET** | | **Cause of death** |
| --- | --- | --- | --- | --- | --- | --- | --- | --- |
|  |  | **Glottic/subglottic**  **Stenosis** | **PET Pattern**  **(0=focal,1=diffuse)** | **Airway-SUVmax** | **Airway-TLG** | **RP related organs** | **Other findings** |  |
| 1/F/44 | 4 | Y | 1 | 6.1 | 119.56 | Costicartilages | Pulmonary inflammation | Lymphoma, breast cancer |
| 2/M/29 | 1 | Y | 1 | 3.23 | 65.91 | Left auricle | ／ | Sudden death probably due to glottis narrowing |
| 3/M/41 | 4 | Y | 0 | 4.54 | 87.80 | Auricle，costicartilages | Pulmonary inflammation | Laryngocarcinoma |
| 4/M/64 | 120 | N | 0 | 2.05 | 47.63 | / | Plumonary inflammatory nodule | Pulmonary infection |
| 5/M/21 | 1 | N | 0 | 3.42 | 28.86 | / | Pulmonary lymphoma | Lymphoma |
